# Effects of health behaviour change intervention through women's self-help groups on maternal and newborn health practices and related inequalities in rural india: A quasi-experimental study

**DOI:** 10.1016/j.eclinm.2019.10.011

**Published:** 2019-11-20

**Authors:** Avishek Hazra, Yamini Atmavilas, Katherine Hay, Niranjan Saggurti, Raj Kumar Verma, Jaleel Ahmad, Sampath Kumar, P.S. Mohanan, Dileep Mavalankar, Laili Irani

**Affiliations:** a Population Council, New Delhi, India; b Bill & Melinda Gates Foundation, New Delhi, India; c Bill & Melinda Gates Foundation, Seattle, United States; d Rajiv Gandhi Mahila Vikas Pariyojana, Uttar Pradesh, India; e Indian Institute of Public Health, Gujarat, India

**Keywords:** Self-help groups, Health behaviour change intervention, Maternal health, Newborn health, Inequality

## Abstract

**Background:**

Despite the health system efforts, health disparities exist across sub-populations in India. We assessed the effects of health behaviour change interventions through women's self-help groups (SHGs) on maternal and newborn health (MNH) behaviours and socio-economic inequalities.

**Methods:**

We did a quasi-experimental study of a large-scale SHG program in Uttar Pradesh, India, where 120 geographic blocks received, and 83 blocks did not receive health intervention. Data comes from two cross-sectional surveys with 4,615 recently delivered women in 2015, and 4,250 women in 2017. The intervention included MNH discussions in SHG meetings and community outreach activities. The outcomes included antenatal, natal and postnatal care, contraceptive use, cord care, skin-to-skin care, and breastfeeding practices. Effects were assessed using multilevel mixed-effects regression adjusted difference-in-differences (DID) analysis adjusting for geographic clustering and potential covariates, for all, most-marginalised and least-marginalised women. Concentration indices examined the socio-economic inequality in health practices over time.

**Findings:**

The net improvements (5–11 percentage points [pp]) in correct MNH practices were significant in the intervention areas. The improvements over time were higher among the most-marginalised than least-marginalised for antenatal check-ups (DID: 20pp, *p*<0•001 versus DID: 6pp, *p* = 0•093), consumption of iron folic acid tablets for 100 days (DID: 7pp, *p* = 0•036 versus DID: -1pp, *p* = 0•671), current use of contraception (DID: 12pp, *p* = 0•046 versus DID: 10pp, *p* = 0•021), cord care (DID: 12pp, *p* = 0•051 versus DID: 7pp, *p* = 0•210), and timely initiation of breastfeeding (DID: 29pp, *p* = 0•001 versus DID: 1pp, *p* = 0•933). Lorenz curves and concentration indices indicated reduction in rich-poor gap in health practices over time in the intervention areas.

**Interpretation:**

Disparities in MNH behaviours declined with the efforts by SHGs through behaviour change communication intervention.

## Research in context

### Evidence before this study

A systematic literature search using PubMed/MEDLINE, Cochrane Library and Popline databases employed keywords such as “self-help groups” or “women's groups,” and “inequality”, along with key terms for “maternal health” or “newborn health” or “child health”. We included quantitative studies published between January 1, 2000 and December 31, 2018 (date of concluding the review). A total of 218 citations were found to be relevant to the subject under review. After removing the duplicates and rejecting the papers after reading the abstracts, 15 papers included the terms “self-help groups” or “community groups” and “maternal health” or “child health”. No previous study systematically assessed the effects of health behaviour change integration intervention through self-help groups (SHGs) by socio-economic disaggregation. Previous studies and systematic reviews of SHGs and health outcomes include findings about changes in maternal and child health outcomes and individual empowerment. While available literature document the positive effects of health behaviour change interventions through women's SHGs on health outcomes, there is a paucity of evidence on reduced inequality in health care practices.

### Added value of this study

This study is part of a community mobilisation project utilising a large SHG program in Uttar Pradesh, India's most populous state, where 120 geographic blocks with SHGs received health behaviour change interventions. The activities included health discussions in SHG meetings, community outreach activities to address gender and social norms, and SHGs established linkages with the health system. There were 83 blocks with SHGs that did not receive health behaviour change intervention and served as a comparison group. The evaluation involved two cross-sectional surveys in 57 sampled blocks from 20 districts with eligible women (age 15–49 years, currently married, given birth in the past 12 months) in both 2015 and 2017. The study notes significant positive changes in maternal and newborn health practices in the intervention areas, compared to the comparison areas. The improvements over time were higher among the most marginalised women, compared to the least marginalised. This is the first large observational study, using a large-scale non-government SHG platform, documenting the effects of health behaviour change interventions on health practices in most- and least marginalised populations in India.

### Implications of all the available evidence

We demonstrate that health behaviour change integration intervention through SHGs not only helps improve maternal and newborn practices, but also reduces disparities between most- and least marginalised populations for such practices. The most marginalised women benefited more through greater program coverage. With the Indian government's huge network of SHGs under its National Rural Livelihood Mission, and focus on ending preventable maternal and newborn deaths, such integration of health messages through the women's SHGs could be a promising approach. The study contributes to the literature, highlighting the fact that microfinance-based community organisations can be effectively used to create an enabling environment for appropriate health information to affect health practices and access to health services, and thereby reduce health inequalities in India, as well as globally.

## 1 Introduction

High maternal and infant mortality in India, particularly in its northern states remain a matter of concern, although substantial improvement has been observed in recent years. According to the latest estimates from the sample registration system, the national maternal mortality ratio has reduced by 22 percent [1], and infant mortality rate has reduced by 21 percent [2], from 2011 to 2016. Substantial disparities in health indicators exist, both between and within states in the country, however, with northern states such as Uttar Pradesh, Haryana and Bihar with the highest burdens [3, 4]. There is an inverse relationship between household socio-economic status and health status [5, 6]; the rural and marginalised poor are most vulnerable to negative health outcomes and contribute more to state and national death rates [1, 2].

It is recognised that most neonatal deaths that occur at home can be prevented by evidence-based clinical services and behaviours [2, 7]. Literature indicates behaviours that have notable impacts on neonatal mortality: Prevention and management of hypothermia, kangaroo mother care, timely initiation of breastfeeding, clean cord care, and safe delivery practices [8], [9], [10]. The key causes of maternal death include postpartum haemorrhage, sepsis, abortion, as well as indirect causes comprising more than a quarter share of total causes of death [11, 12]. Safe delivery practices along with antenatal check-ups and treatment of complications during pregnancy, delivery or postpartum help identify potential complications in time and their effective management, saving the lives of mothers. Persistent disparities in maternal and newborn health service coverage exist throughout India, whether economic [13, 14], social [15] or spatial [16], and these result in neonatal and maternal health practice inequalities [17].

Women's microfinance-based self-help groups (SHGs) in marginalised populations is a promising strategy for empowering women [18] and improving their health outcomes [8, [19], [20], [21], [22]]. A significant proportion of maternal and newborn mortalities and morbidities, and especially those among women unable to access health facilities or outreach services, could be reduced by developing and expanding coverage by community-based platforms, such as SHGs, and building strong linkages with the lowest levels of the state health system such as frontline health workers (FLWs) [23, 24]. Networking SHGs and relevant health and other community institutions also expands women's social networks and social capital, fosters solidarity and mutual learning, and creates many opportunities for women's empowerment, enabling not only their self-advocacy for services, but accountability [25].

This study utilised a large pool of SHGs run by a non-government entity for poverty reduction and women's empowerment, espousing the fundamental values of unity, self-reliance, inclusiveness, support and volunteerism. Women's SHGs that include marginalised population sub-groups offer an opportunity to reduce inequalities and fortify the health system [26]. Literature shows that health interventions through women's groups have improved maternal and newborn health behaviours [8, [19], [20], [21], [22], [27], [28], [29], [30], [31], [32]], and adequate population coverage by SHGs and participation of at least one third of pregnant women in a catchment area in groups reduce neonatal mortality [8]. Most studies demonstrate that participatory learning and action through women's SHGs is an effective mechanism for engaging women in health discussion and enabling correct health behaviours. Women's education, social caste and economic stratum play important role in women's health, however [26]. A paucity of studies investigate whether health interventions through a large volume of women's SHGs with diverse geographic coverage is effective, and whether such interventions affect all segments of a community in a similar manner, or whether effect varies by household marginalisation status.

We assessed the effects of health behaviour change interventions through women's SHGs on maternal and newborn health behaviours in the overall sample of women as well as those in different socio-economic strata. In this paper we hypothesised that health behaviour change interventions through women's groups would help reduce inequalities in accessing health care services and practice of healthy behaviours in rural communities of northern India.

## 2 Methods

### 2.1 Intervention setting

This study is an evaluation of a health behaviour change intervention integration within SHGs, formed and managed by a large microfinance-based women's group, the *Rajiv Gandhi Mahila Vikas Pariyojana* (RGMVP) in Uttar Pradesh. RGMVP, a non-government organisation, that work to strengthen the community institutions of the poor [18], is built on a self-help, voluntary, three-tiered approach, working at scale with about 1•7 million poor women across 49 districts of the state. RGMVP uses a participatory approach for identifying the most disadvantaged families, who usually belong to lower social caste, from poor families and live in small or isolated hamlets and villages. The SHGs (first tier) at the village level are federated into village organisations (VOs) (second tier) representing 150 to 250 women from 10 to 20 SHGs. VOs, in turn, are federated into block organisations (third tier) of 5000 to 7000 women [30]. RGMVP has been organising poor rural women to break the vicious circle of poverty through these women's access to microfinance and social capital through SHG membership. RGMVP also aims to empower women by making them aware of their entitlements and demand them from the Government, including local governing bodies like *Panchayats*. Each SHG meets weekly to discuss microfinance and livelihood issues and maintains a register. RGMVP identified 120 blocks, by their operational principles for the health behaviour change intervention through SHGs and their federations.

The primary intervention included maternal and child health information dissemination in SHG meetings by trained peer educators, building community norms for behaviour change through a set of community outreach activities including home visits, community meetings, community events such as *Godhbharai* (a ceremony to celebrate pregnancy) and *Annaprasan Diwas* (a ceremony typically for a child of six to eight months age to initiate complementary feeding, organised at the *Anganwadi* centre every month, on a fixed date) with active facilitation and arrangement by VOs, and use of audio visual aids such as health videos.

SHGs, as social platforms, work as vehicles for behaviour change communications as well as improving health system access through community health workers. To implement the health intervention, SHGs were asked to select one member to be a community health volunteer, called *Swasthya Sakhi*, to work as a peer-educator. All selected *Swasthya Sakhis* were trained on key maternal and newborn health behaviours. Each *Swasthya Sakhi,* in a weekly meeting each month, provided information on healthy practices, encouraged SHG members to participate in discussion, and stressed the importance of those correct practices to save both mothers and children. The maternal health component included identification of danger signs and referrals, a complication readiness plan, family planning, and promotion and facilitation of antenatal and postnatal care (ANC and PNC). The newborn health component included thermal care (skin-to-skin care or kangaroo mother care and delayed bathing), breastfeeding, prevention of infection (cord care), identification of newborn danger signs and referral. The details of the intervention activities are presented in Table 1.

**Table 1:** Activities in the comparison and intervention area.

Activities	Comparison area	Intervention area
SHG meetings	SHG leaders conducted up to four meetings every month to discuss microfinance and livelihood issues	Same as comparison area
Health discussion in SHG meetings	Health was not part of discussion in SHG meetings	Health was part of discussion in at least one weekly meetings in a month. Discussion on MNH issues was done by a trained community health volunteer, who worked as a peer-educator. They provided information on healthy MNH practices, engaged all the members to participate in discussion, and highlighted the importance of the correct health practices to save mother and child in their families
Home visits	FLWs of the government health system visited women at their home during pregnancy and after delivery	FLWs of the government health system visited women at their home during pregnancy and after delivery
		Additionally, *Swasthya Sakhi* or SHG members or VO members made home visits, separately as well as jointly with FLWs, during women's pregnancy and after delivery, to provide information to women as well as their family members about correct MNH practices
Distribution of leaflets and letters containing MNH information to target women	No leaflets containing health messages were given	Two types letters – welcome letter (*Subhkamna Patra)* and congratulatory letter (*Badhai Patra)*, and leaflets were developed. These letters and leaflets had pictorial information regarding required care during pregnancy, delivery and after delivery, danger signs that require attention during pregnancy, delivery and after delivery, neonatal danger signs, and correct newborn health practices. The letters were signed by a VO member and SHG members gave the welcome letter to families of pregnant women and congratulatory letters to families of recently delivered women. The leaflets were given to the family members of the pregnant and recently delivered women.
*Purwa* level meeting	Not done	The leaders of the VOs with the help of a designated woman, internal social capital, conducted meetings at village or hamlet (*Purwa*) level to discuss MNH issues with larger community that included non-SHG women as well as men
Night meeting	Not done	The leaders of the VOs with the help of internal social capital conducted meetings with SHG members in the night so that women, who missed meetings in day time due to their engagement in household and outside work, could attend these meetings and participate in discussion MNH issues
Village Health and Nutrition Day	On this day, Anganwadi Workers (AWWs) and Accredited Social Health Activists (ASHAs) mobilised the villagers, especially pregnant women and new mothers along with children to gather at the nearest Anganwadi Centre, where they are provided with integrated health solutions as per their needs.	Same as in the comparison area
		Additionally, as part of the intervention component of greater linkages with local health functionaries, SHG members motivated and accompanied women and their children to the Anganwadi centres on the Village Health and Nutrition Days to receive health services
		SHG members helped women provide health information through FLWs on this occasion
*Godhbharai* event	AWWs organised this event at the Anganwadi Centre as per their convenience, although it is mandated to organise once in every month.	SHGs with support from VOs and jointly with AWWs organised this event every month, where all pregnant women in the catchment area were called to celebrate the pregnancy and were felicitated with small gifts such as bowls, piggy bank (*gullak)*, fruits etc. The internal social capital discussed health messages with all attendees, which comprised of elder female family members including mothers-in-law of the target women. Pregnant women, whose pregnancy had already been celebrated through this event, were also encouraged to participate in the subsequent *Godhbharai* events, so that they receive health information multiple times
*Annaprasan diwas*	AWWs organised this event at the Anganwadi Centre as per their convenience, although it is mandated to organise once in every month.	SHGs with support from VOs and jointly with AWWs organised this event every month, where all mothers with children of age 6–8 months in the catchment area were called. The event comprised of following activities: discussion around child health and nutrition, demonstration of complementary food preparation and initiation of complementary food to children aged 6–8 months.
Health video shows	Not done	Videos on MNH issues was developed as part of the intervention and was shown at various places such as *Purwa* meetings, at the Anganwadi Centres on the Village Health and Nutrition Days, at meetings conducted in night.

### 2.2 Control setting

In 83 comparison blocks, RGMVP groups feature regular microfinance activities including savings, internal lending and loan repayment. Unlike in the intervention blocks, maternal and neonatal health were not part of the comparison block SHG discussions. Communities received normal health services from FLWs employed by the public health system (Table 1).

### 2.3 Study location and population

**Fig. 1:**
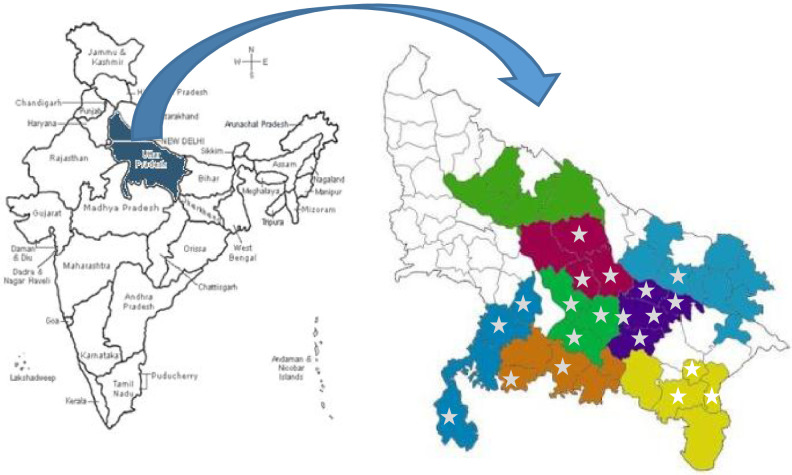
Study area. **Note:** Shaded area indicates the districts with presence of the SHGs under study, the eight colour shades indicate the eight zones (called Community Resource Development Centers) of the program functioning, and * indicates the sampled districts for evaluation.

**Fig. 2:**
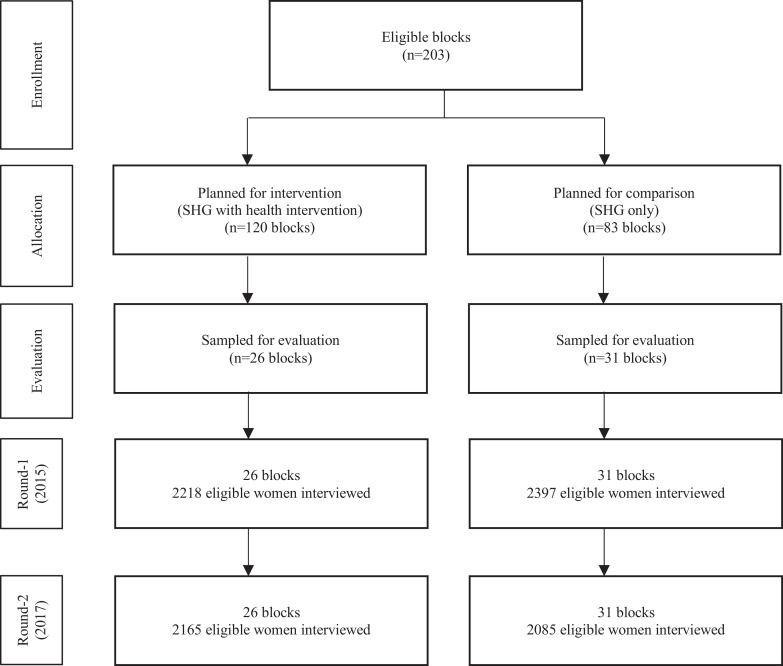
Study sample.

The study was conducted in Uttar Pradesh, the most populous state of northern India, with a population of 199•8 million with 78 percent of them live in rural areas in 2011 [33]. In each wave of data collection, eligible women, who were currently married, 15–49 years of age, and had given birth in the 12 months prior to the survey, were selected cross-sectionally. The study used data from 4615 eligible women in 2015 and 4250 eligible women in 2017, sampled from 57 blocks in 20 districts (Fig. 1). The sample sizes were estimated based on the prevalence of primary outcomes prior to round 1 survey as per available sources, at least 7 percentage points (pp) expected change after intervention, power to detect those differences at 0•85, level of significance at 0•05, and design effect 2. Eligible women were sampled from the same blocks in each survey round (Fig. 2).

### 2.4 Evaluation approach

The evaluation assessed the effects of an intervention using a quasi-experimental design. The present study involves information collected from eligible women from SHG households (at least one woman from the household is a SHG member) in two types of blocks: (1) with both SHGs and a health behaviour change intervention integrated within the SHGs (intervention blocks), and (2) SHGs without a health behaviour change intervention (comparison blocks).

A multi-stage sampling design was used to select the study participants. In the first stage, all intervention blocks were arranged in ascending order by percent of scheduled caste or scheduled tribe (SC/ST) population, a critical parameter for development. The required number of blocks was then selected by systematic random sampling. In the second stage, *Gram Panchayats* (GPs), India's lowest administrative units, were selected to include diverse GPs in terms of the proportion of population covered by SHG in the sample. GPs with less than five percent coverage and greater than 60 percent coverage were excluded from the sampling frame to remove the outliers. The remaining GPs were divided into three strata: 5 to 15 percent, 16 to 30 percent and 30 to 60 percent population coverage by SHGs, and the required number of GPs were drawn equally from each of the three strata. Mapping and house listings of the selected GPs identified eligible households from all SHGs to provide eligible women for a sampling frame in each survey round. All eligible women were approached for interview. Once the required numbers of intervention blocks were selected, corresponding comparison blocks were selected based on simple matching by two criteria: 1) within the same district (in case of non-availability of comparison block in the same district, a geographically adjacent district was chosen) to reduce the effect of socio-cultural diversity between study geographies, if any, and 2) similar proportion of SC/ST population in the selected intervention blocks. Matched comparison blocks were chosen independently for each selected intervention block. The average proportion of SC/ST population in the selected intervention and comparison blocks were similar (27•2% and 29•0% respectively). The GP and household selection strategy for comparison blocks followed the same criteria as for the intervention blocks.

Questionnaires were administered in the local language, Hindi. Information on housing characteristics was collected from the household heads, and information on maternal and newborn health knowledge and practices were collected from the sampled eligible women. Written informed consent was obtained from literate women, while thumb impression or oral consent were provided by women with no formal education. Respondents were free to decline an interview, at any time. One copy of the consent form was given to the respondent, for her record. Data were collected using the computer-assisted personal interview technique, by trained female research investigators, extensively trained by the research team for 15 days on both the technical and ethical aspects of data collection. Data entry for the study utilised the Census and Survey Processing System (CSPro) of the United States Census Bureau and ICF International. The study protocol and its survey tools were reviewed and approved by the Institutional Review Board of the research institution.

### 2.5 Measures

The primary outcomes included in the study are:

*Reproductive and maternal health practices:* At least four ANC visits, at least three ANC check-ups, consumption of 100 or more iron folic acid (IFA) tablets, institutional delivery, PNC check-up within first seven days of delivery, and current use of any contraceptive method. These indicators were based on the following questions asked of women: 1) number of ANC visits by the respondent during her last pregnancy, 2) whether ANC check-ups such as blood test, blood pressure measurement and abdominal examination were made during her ANC visits (each option coded as yes/no), 3) number of IFA tablets consumed during the pregnancy, 4) place of last delivery (health facility/home), 5) number of PNC check-ups within seven days of delivery, and 6) whether respondent was currently using any contraceptive method (yes/no).

*Newborn health care practices:* Clean cord-care to prevent cord infection, skin-to-skin care to keep the newborn warm, timely initiation of breastfeeding, and exclusive breastfeeding. Outcomes were computed based on women's reported responses, and information used to create these outcome measures included: 1) what was done to prevent cord infection of the newborn (‘nothing was applied on cord stump’ was treated as correct practice), 2) what was done to keep the newborn warm (spontaneous response of ‘skin-to-skin care/ kangaroo mother care’ or ‘yes’ response to direct question on practice of skin-to-skin care/ kangaroo mother care was treated as correct practice), 3) amount of time after delivery newborn was placed on mother's chest for breastfeeding (‘within an hour’ was treated as correct practice), 4) whether child was breastfed the day or night preceding survey (yes/no) and at what age water, or other milk or semi-solid/solid food was given to newborn (if newborn was breastfed the previous day or night and nothing except mother's milk was given, it was treated as correct practice). The computation of newborn health outcomes was restricted to women with children less than six months of age (0 to 179 days).

The survey rounds (round 1 in 2015 versus round 2 in 2017) and study arm (intervention versus comparison) were the key independent variables. Degree of marginalisation, another independent variable, was computed through a composite index of women's education, caste and household wealth index to assess the effect of health intervention on health practices by marginalisation status. The wealth index (lowest, lower, middle, higher, highest) was treated as a proxy to household income or expenditure [34, 35]. For the composite index of marginalisation, each of the three variables of education (no formal education=0, else=1), caste (SC/ST=0, else=1) and wealth index (lowest or lower wealth quintile=0, else=1) were dichotomised and an additive score from 0 to 3 was computed. Three categories of marginalisation were created: most marginalised (score 0), somewhat marginalised (score 1 or 2) and least marginalised (score 3). The most marginalised category includes women with no formal education, SC/ST, and from households in the lowest or lower wealth quintiles, while the least marginalised category includes women with formal education, from other social castes/classes, and belonging to households with middle or higher/highest wealth quintiles.

To examine if the program reached the most- and least marginalised women in a similar manner, the following information was utilised: i) number of SHG meetings attended in last one month, ii) whether called by an SHG member to attend a meeting with health discussion (yes/no), iii) whether attended any *Godhbharai* event during pregnancy (yes/no), iv) whether seen any health videos in last three months (yes/no), v) whether attended any *Purwa* meeting in last three months (yes/no), vi) whether attended any night meeting in the last three months (yes/no), vii) whether anyone from SHG accompanied respondent during any of her ANC care visits, viii) whether respondent discussed delivery plan with anyone from SHG (yes/no), and ix) whether anyone from SHG accompanied respondent to health facility for delivery (yes/no). Information on exposure to intervention activities were collected from women in the intervention area only, and except meeting attendance, no information was available in round 1 data.

### 2.6 Statistical analysis

The differences between the two study groups were tested by using ordinary least-squares (for continuous variables) or logit (for categorical variables) regression models at each survey round. The differential effects in healthy practices among women from SHG households in the intervention and comparison areas over time were estimated using multilevel mixed-effects regression adjusted difference-in-differences (DID) analysis. Estimates were adjusted for clustering, considering blocks as a random effect. Variables included for adjustment in the regression model were women's education, parity, caste, family structure, wealth quintile, mass media exposure, respondent's SHG membership and duration of household SHG membership. Selection of covariates was based upon empirical exploration and literature. The empirical exploration included examination of the differences between the study arms at both the survey rounds for all potential covariates having a relationship either with use of intervention or with outcome measures. All such variables, for which significant differences between the study arms were observed either in round 1 or in round 2, were considered as covariates in the regression model. Further, based on literature, women's education, although were similar between the study arms, was included in the regression. The wealth index was developed through principal component analysis using information of housing characteristics and household assets. The effect sizes among the most- and least marginalised were also computed using multilevel mixed-effects regression adjusted DID analysis, accounting for clustering effect. Bi-variate analysis examined women's exposure to health intervention activities by their marginalisation status.

Women's practice of healthy behaviours in the two study arms were compared between the two survey rounds using concentration index and Lorenz curves using wealth score as the key parameter. Concentration index is an appropriate measure of health inequality, since it meets the three basic requirements of a health inequality index: It reflects the socio-economic dimension of inequalities in health, reflects the experiences of the entire population, and is sensitive to changes in population distribution across socio-economic groups [36]. The concentration index is defined as twice the area between the concentration curve and the diagonal, ranging from −1 to +1. The value of the index measures the severity of socio-economic inequality: The larger the absolute value of index, the greater the disparity. The Lorenz curves provide a clear visual depiction of socio-economic inequalities of health outcomes. The concentration indices were computed and Lorenz curves were drawn for maternal health outcomes—ANC visits, check-ups, consumption of IFA tablets, institutional delivery, PNC check-ups and use of contraceptive method—dependent upon supply of services or access to health facilities. Lorenz curves were not drawn for newborn health outcomes—cord care, thermal care and breastfeeding practices—dependent on women's learning and practices, and less dependent on supply side factors [20]. All analyses employed Stata version 13•0 (StataCorp, College Station, TX, USA).

## 3 Results

The socio-demographic characteristics of sampled women interviewed at the two rounds of survey are presented in Table 2. In both rounds, the average respondent age was around 26 years with parity three across the study arms. Women's education was similar in both study arms, in both rounds; around one third of respondents in round 1 and around one fourth in round-2 had no formal education. About one fifth of women interviewed were working in the 12 months preceding the round 1 survey, while less than one tenth had been working in round 2. Women's exposure to mass media, reading to a newspaper, listening to radio or watching television, was limited; however, significantly higher proportion of women, at both survey rounds, from the intervention area had exposure to any of those media. Around 44 to 50 percent of respondents were from nuclear families at round 1, while the proportions of respondents from nuclear families in round-2 were from 28 to 36 percent. More than 90 percent of respondents at each round in both study arms were Hindu. The proportion of SC/ST women interveiewed was higher in the comparison arm (56%) than the intervention arm (46%) in round 1, and similar difference was noted at round 2 (50% versus 41%). A significantly higher proportion of respondents in the compariosn arm than the intervention arm were from the lowest two wealth quintiles in both survey rounds. More than 90 percent of women reported at least one contact with any FLW during pregnancy, and around 50 to 60 percent of women had contact with a FLW at anytime within seven days of delivery; no significant difference between study arms in any survey rounds was found. A significantly higher proportion of respondents from the comparison areas than the intervention areas were SHG member in each survey round. Women from the intervention area were from households with relatively longer SHG association compared to the comparison area, both at round 1 (36 months versus 16 months) and round 2 (73 months versus 46 months). A higher proportion of women in the comparison area than the intervention area were in the most marginalised category in both survey rounds.

**Table 2:** Characteristics of study participants over time, 2015–17.

	Round-1 (2015)			Round-2 (2017)		
	Intervention area	Comparison area	p-value	Intervention area	Comparison area	p-value
Number of women	2218	2397	–	2165	2085	–
Women's age in years, mean (S.D.)	26•2 (4•48)	26•2 (4•47)	0•996	25•9 (4.66)	25•8 (4•73)	0•734
Women's parity, mean (S.D.)	2•8 (1•74)	2•9 (1•72)	0•596	2•7 (1•68)	2•8 (1•74)	0•007
Sex of the indexed child is male,%	52•2	51•3	0•501	52•4	50•3	0•149
Women's education,%						
No formal education	38•5	39•3	0•811	26•1	29•6	0•230
Up to primary	16•9	18•7	0•239	17•9	18•5	0•734
Up to secondary	28•3	26•9	0•422	31•1	30•2	0•642
Higher secondary and above	16•4	15•1	0•627	21•7	24•9	0•334
Women currently working,%	18•7	21•7	0•254	6•7	8•7	0•109
Mass media exposure,%	32•7	22•4	0•006	51•2	41•3	0•028
Nuclear family structure,%	43•6	49•7	0•165	28•0	35•8	0•015
Hindu religion,%	92•2	93•3	0•555	91•4	92•9	0•479
Scheduled caste/tribe,%	45•6	55•7	0•008	41•3	50•0	0•038
Wealth quintiles,%						
Lowest	19•0	24•4	0•162	14•6	23•9	0•021
Lower	19•2	24•9	0•001	18•9	23•9	0•005
Middle	22•1	20•5	0•391	21•4	20•6	0•589
Higher	20•7	19•2	0•476	23•0	18•0	0•034
Highest	19•2	11•0	0•001	22•2	13•6	0•003
Any contact with FLW during pregnancy,%	93•2	94•1	0•560	96•5	96•4	0•863
Any contact with FLW within 7 days of delivery,%	52•3	48•1	0•058	62•6	59•4	0•225
Respondent herself is an SHG member,%	69•2	75•3	0•064	47•5	56•1	0•008
Household's SHG membership duration in months, mean (S.D.)	36•1 (32•66)	15•5 (10•65)	<0•001	73•3 (35•6)	45•7 (13•90)	<0•001
Women in the most marginalised category,%	13•6	16•1	0•290	7•8	11•5	0•024
Women in the least marginalised category,%	29•8	19•9	0•003	37•9	25•5	0•001

**Note:** Differences in groups at baseline and endline were tested by using ordinary least-squares regression models (continuous variables) or logit regression models (categorical variables), adjusting for clustering effect at the block level. S.D: standard deviation; mass media exposure is computed based on exposure to either of the three print or electronic media—newspaper, radio and television.

Multilevel mixed-effects regression-adjusted DID estimates show statistically significant improvement in six outcomes that increased by net 5 to11 pp over time in SHG families in the intervention areas, as compared to SHG families in comparison areas (Table 3): At least four ANC visits (DID 5pp, *p* = 0•004), at least three tests or examinations during pregnancy (DID 8pp, *p*<0•001), PNC check-up within a week of delivery (DID 5pp, *p* = 0•013), current use of any contraceptive method (DID 11pp, *p*<0•001), clean cord care (DID 7pp, *p* = 0•004), and timely initiation of breastfeeding (DID 6pp, *p* = 0•047).

**Table 3:** Effects of health intervention on maternal and newborn health behaviours, 2015–17.

Indicator	Intervention area		Comparison area		Net change [95% Confidence Interval]	p-value
	Round-1 (*N* = 2218)	Round-2 (*N* = 2165)	Round-1 (*N* = 2397)	Round-2 (*N* = 2085)		
***Maternal health practices***						
At least 4 ANC visits	17•2	45•7	14•3	37•6	5•2* [1•6, 8•7]	0•004
At least 3 tests during ANC visits^a^	37•7	66•2	35•2	55•4	8•3* [4•4, 12•2]	<0•001
Consumption of 100 or more IFA tablets during pregnancy	13•0	19•1	8•7	12•9	1•9 [−0•9, 4•8]	0•186
Institutional delivery	83•1	87•8	79•7	85•1	−0•7 [−3•7, 2•3]	0•650
Postnatal check-up within a week of delivery	19•5	34•3	20•0	30•3	4•6* [1•0, 8•2]	0•013
Current use of any contraceptive method	38•0	45•4	45•6	41•7	11•2* [7•0, 15•4]	<0•001
***Newborn health practices***						
Clean cord care (0–5 months)	19•2	38•9	12•2	24•6	7•4* [2•3, 12•4]	0•004
Skin-to-skin care (0–5 months)	27•8	44•1	11•9	24•5	3•7 [−1•6, 9•0]	0•171
Timely initiation of breastfeeding (0–5 months)	68•4	72•8	64•1	62•8	5•8* [0•1,11•5]	0•047
Exclusive breastfeeding^b^ (0–5 months)	69•6	65•4	65•0	62•7	−1•8 [−11•1, 7•4]	0•697

**Note:** Round 1 survey was conducted in 2015 and round 2 survey was conducted in 2017. Net change was estimated using mixed-effect multilevel regression adjusted difference-in-differences (DID) analysis and adjusted for women's education, parity, caste, family structure, wealth quintile, mass media exposure, respondent's membership in groups and duration of household's SHG membership in the regression analysis, accounting for clustering effect at block level.

a Includes blood test, blood pressure measurement, and abdominal examination.

b This indicator was calculated as proportion of infants 0–5 months of age who were fed exclusively with breast milk, based on last 24 h recall. Calculation included only those women who were interviewed in the same months of data collection in the two rounds of survey to control for the seasonality bias.

⁎ refers *p*<0•05.

Findings presented in Table 4 indicate that improvements in maternal and newborn health practices were substantially higher among the most marginalised than least marginalised women, specifically for at least three tests or examinations during ANC visits (DID: 20pp, *p*<0•001 versus DID: 6pp, *p* = 0•097), consumption of 100 or more IFA tablets during pregnancy (DID: 7pp, *p* = 0•036 versus DID: −1pp, *p* = 0•671), current use of contraceptive methods (DID: 12pp, *p* = 0•046 versus DID: 10pp, *p* = 0•021), clean cord care (DID: 12pp, *p* = 0•051 versus DID: 7pp, *p* = 0•210), and timely initiation of breastfeeding (DID: 29pp, *p* = 0•001 versus DID: 1pp, *p* = 0•933).

**Table 4:** Effect of the health intervention on select maternal and newborn health indicators for least marginalised and most marginalised groups, 2015–17.

	Least marginalised						Most marginalised					
	Intervention area		Comparison area		Net change [95% Confidence Interval]	p-value	Intervention area		Comparison area		Net change [95% Confidence Interval]	p-value
	Round-1	Round-2	Round-1	Round-2			Round-1	Round-2	Round-1	Round-2		
***Maternal health practices***												
At least 4 ANC visits	24•1	56•2	19•4	48•6	3•0 [−4•5, 10•4]	0•434	8•3	33•0	5•1	26•6	3•2 [−5•2, 11•6]	0•452
At least 3 tests during ANC visits^a^	50•3	74•5	49•2	66•9	6•4 [−1•1, 14•0]	0•093	22•8	55•9	21•4	34•7	19•9* [9•0, 30•7]	**<0•001**
Consumption of 100 or more IFA tablets during pregnancy	18•2	22•9	12•8	18•9	−1•4 [−7•7, 4•9]	0•671	9•1	20•9	3•0	7•5	7•3* [0•5, 14•1]	**0•036**
Institutional delivery	91•1	93•3	88•2	93•1	−2•8 [−7•1, 1•6]	0•212	72•4	83•9	69•4	75•4	5•4 [−5•4, 16•3]	0•325
Postnatal check-up within a week of delivery	25•1	44•5	22•4	36•3	5•6 [−1•9, 13•1]	0•143	14•1	20•2	14•5	18•8	1•7 [−7•5, 18•8]	0•718
Current use of any contraceptive method	43•2	52•1	48•7	48•0	9•7* [1•4, 17•9]	**0•021**	33•6	36•9	40•8	31•8	12•3* [0•2, 24•4]	**0•046**
***Newborn health practices***												
Clean cord care (0–5 months)	19•4	38•4	12•0	24•5	6•5 [−3•7, 16•7]	0•210	16•7	31•9	14•2	17•0	12•3 [−1•2, 25•9]	**0•051**
Skin-to-skin care (0–5 months)	27•1	43•2	14•8	27•3	3•6 [−7•2, 14•4]	0•512	28•0	42•9	7•8	20•0	2•6 [−11•5, 16•7]	0•717
Timely initiation of breastfeeding (0–5 months)	67•9	65•4	64•0	61•0	0•5 [−10•8, 11•8]	0•933	63•8	85•2	67•0	59•5	28•9* [12•5, 45•4]	**0•001**
Exclusive breastfeeding^b^ (0–5 months)	71•3	63•3	75•3	55•6	11•8 [−6•1, 29•7]	0•198	61•7	69•9	63•5	62•9	8•8 [−13•5, 31•1]	0•439

**Note:** Round 1 survey was conducted in 2015 and round 2 survey was conducted in 2017. Net change was estimated using mixed-effect multilevel regression adjusted difference-in-differences (DID) analysis and adjusted for women's education, parity, caste, family structure, wealth quintile, mass media exposure, respondent's membership in groups and duration of household's SHG membership in the regression analysis, accounting for clustering effect at block level.

a Includes blood test, blood pressure measurement and abdominal examination.

b This indicator was calculated as proportion of infants 0–5 months of age who were fed exclusively with breast milk, based on last 24 h recall. Calculation included only those women who were interviewed in the same months of data collection in the two rounds of survey to control for the seasonality bias.

⁎ refers *p*<0•05.

**Fig. 3:**
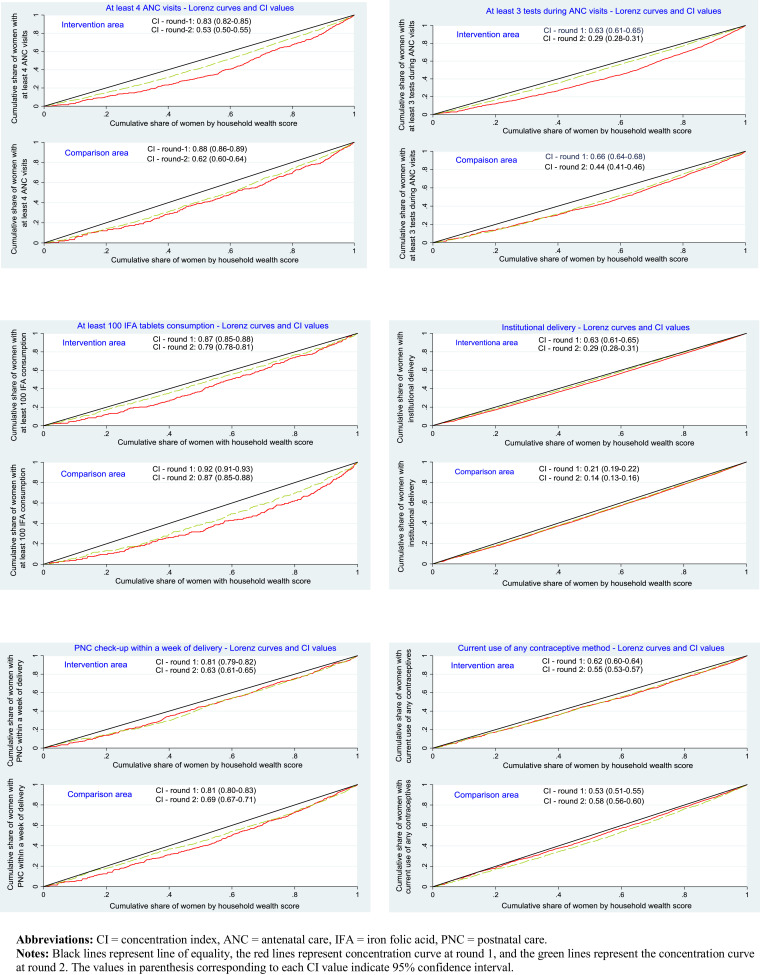
Lorenz curve and concentration index for maternal health behaviours in intervention and comparison blocks over time. **Abbreviations:** CI = concentration index, ANC = antenatal care, IFA = iron folic acid, PNC = postnatal care. **Notes:** Black lines represent line of equality, the red lines represent concentration curve at round 1, and the green lines represent the concentration curve at round 2. The values in parenthesis corresponding to each CI value indicate 95% confidence interval.

Lorenz curves were drawn to represent the influence of wealth inequalities on maternal and neonatal health outcomes. The concentration curves, as well as the values of concentration indices in the two rounds, indicate rich-poor gap reduction in health practices over time in the intervention areas (Fig. 3). The black lines represent line of equality, the red lines represent concentration curve at round 1, and the green lines represent the concentration curve at round 2. The rich-poor gap in accessing services including at least three tests or examinations during ANC visits, institutional delivery and PNC check-up reduced notably over time in the intervention area, compared to the comparison area. For example, the value of the concentration index for at least three tests during pregnancy reduced from 0•63 in round 1 to 0•29 in round 2 in the intervention area compared to a reduction of 0•66 to 0•44 in the comparison area. Similarly, in the intervention area the concentration index for institutional delivery reduced from 0•63 in round 1 to 0•29 in round 2, and the values corresponding to PNC check-up reduced from 0•81 to 0•63 over a period of two years.

Findings on women's exposure to the intervention activities and support from the SHG members during pregnancy and after delivery revealed that significantly a higher proportion of women in the most marginalised category (59%) than in the least marginalised category (46%) attended a SHG meeting in the month prior to the survey (*p* = 0•003) (Table 5). Similarly, 41 percent of most marginalised women, as compared to 26 percent least marginalised women, reported invitation to attend a meeting with a health discussion (*p*<0•001). Women's exposure to community outreach activities was low, however, higher among most marginalised than the least marginalised, although statistically not significant. Approximately 30 percent of most marginalised women, as compared to about 22 percent of least marginalised women, discussed their delivery plan with any SHG member (*p* = 0•024) and were accompanied by an SHG member to a health facility for delivery (*p* = 0•032).

**Table 5:** Women's exposure to the health intervention activities by marginalisation status in the intervention area, 2017.

Indicator	Least marginalised	Most marginalised	p-value#
Attended SHG meeting in last one month	46•2	58.9	0.003
Called by SHG to attend health meetings	26•3	40.5	<0.001
Attended *Godhbharai* event	12•2	16•1	0•167
Seen health videos in last 3 months	3•7	5•4	0•303
Attended *Purwa* meetings in last 3 months	5•2	6•5	0•496
Attended night meetings in last 3 months	1•7	6•5	<0•001
Accompanied by any SHG member for ANC visits	13•7	13•7	0•976
Discussed delivery plan with any SHG member	22•3	30•4	0•024
Accompanied by any SHG member to health facility for delivery	22•1	29•8	0•032

**Note:** Information on program exposure is not available in the round-1 data and not applicable for the comparison area; # p-values are based on the z-test to compare the two proportions.

## 4 Discussion

The findings from this study indicate that discussions of key maternal and newborn health practices in SHG meetings, along with community outreach activities, help women follow correct health care practices. These results are consistent with findings from other studies demonstrating effects of participatory learning approach through women's SHGs for improving healthy practices [8, 9, 20, [27], [28], [29], 31]. The evidence from this study in a large north Indian state adds to the literature of participatory learning through women's groups helping to further reduce the disparities in maternal and newborn health care practices within socio-economic strata.

Consistent with findings from studies of similar interventions in other parts of the country [20, 31], this study shows significant improvements in health practices in the intervention areas, particularly practices that are one-time point and independent of health care service supplies. In the comparison areas, some maternal and newborn practices also improved, which could be attributed to increased social cohesion and group collectivisation identified in other studies [20]. The results of this study as well as literature reveal that in less developed Indian states, women's exposure to mass media remains low [37], [38], [39], [40]. Further, FLWs are able to reach less than half of households in their catchment areas due to multi-faceted factors, with women residing in small hamlets or from lower socio-economic strata left out [41]. These factors lead to the lack of awareness among the marginalised women about correct health practices along with less contact with FLWs and their limited access to service facilities for health care. The strength of this paper is in its findings that health behaviour change intervention through SHGs are able to improve health practices of the most marginalised women, who are often left out from accessing health information or services. An analysis showed that contact with FLWs among most marginalised women in the intervention area increased in the two years of intervention, compared to similar women in the comparison area. Further, in the intervention area, SHGs played a crucial role in reaching most marginalised women, both engaging them in intervention activities and through the social support, such as accompanying them to health facilities. It is evident that health behaviour change interventions through women's SHGs not only improve healthy practices, but that gains are greater among those more socio-economically disadvantaged.

Although this study's findings indicate strong relationship between health intervention integrated within SHGs and its effect in reducing inequalities in maternal and newborn health care practices, the results of this study should be interpreted cautiously in light of certain study limitations. First, it was not possible to randomise the intervention allocation between blocks, as the implementing organisation decided the blocks for intervention based on their operational appropriateness. Any potential bias in allocation of intervention has been addressed with appropriate statistical models adjusting for geographic clustering, considering blocks as a random effect. Second, the comparison blocks were selected after matching the proportions of SC/ST populations in the intervention blocks. Although, the proportions of SC/ST populations were similar in the sampled intervention and comparison blocks,a significantly higher proportion of respondents were from SC/ST families in the comparison area. Third, the average duration of SHG membership in the intervention area was significantly higher, primarily due to the nature of the intervention. The experiment of health intervention integration within SHGs was planned with groups extant for some time along with newly formed groups, while the SHGs in the comparison areas were mostly newly formed at the time of first round of survey. Both these variables were adjusted, however, through regression analysis and may well not imply potential bias in the results. Another point of caution is that responses are based on self-reporting, which may involve recall bias. To reduce any recall bias for health practices, the study eligibility criteria included women who had given birth in the last year preceding the survey for both the survey rounds. Furthermore, for newborn health practices, outcome measures are restricted to women with children less than 6 months of age, to reduce recall bias.

Health behaviour change intervention within SHGs not only improves positive health practices, but also reduces disparities between most- and least marginalised populations for such practices. This indicates that the poorer are more likely to benefit from health intervention integration within community-based participatory learning programs like SHGs. With the Indian government's huge network of SHGs under its National Rural Livelihood Mission, and focus on ending preventable maternal and newborn deaths, such integration of health messages through women's SHGs could be a promising approach. A similar health integration initiative is being implemented in another large north Indian state, Bihar, through the government-run SHG program. Such interventions help change social and gender norms, through the interactions of SHG members and FLWs, not only with targeted women, but their family members including mothers-in-laws and husbands, through multiple ‘touch-points’ such as home visits and communtiy outreach activities. Further research is required to deduce the mechanisms of impact on health behaviours, however. This study contributes to the literature by emphasising the fact that microfinance-based community organisations can be effectively used to create enabling environments for receiving important health information, for critical health behaviours and access to health services, and thereby reduce health inequalities in India, as well as globally.

## Funding

The Population Council was funded for this evaluation study by the Bill & Melinda Gates Foundation (grant OPP1033910).

## Role of the funding source

YA and KH were from the funder, provided technical inputs in the sampling design. The final decisions on the manuscript content lay with the primary authors from the evaluation team (AH, NS, RKV, JA, LI). The corresponding author had full access to the data used in the study and had final responsibility for the decision to submit for publication.

## Author contributions

AH conceptualized this study; AH, YA, KH, DM, SK, JA, PSM designed the overall study; AH, JA, RKV and LI led the data collection; AH developed the first draft of the manuscript; AH and RKV conducted the data analysis; YA, KH, NS and LI advised on the analysis; NS, LI, RKV and JA critically reviewed the manuscript; SK, PSM, DM and JA provided input to the sections on intervention and implementation design; and all authors read and approved the final manuscript.

## Declaration of Competing Interest

AH, YA, KH, NS, SK, RKV, JA, DM, PSM and LI, no conflicts of interest.

## Appendix Supplementary materials

Image, application 1

Image, application 2
