# Supplementary material for: Effects of health behaviour change intervention through women's self-help groups on maternal and newborn health practices and related inequalities in rural india: A quasi-experimental study
Source: eClinicalMedicine. 2019 Nov 20;18:100198. doi: 10.1016/j.eclinm.2019.10.011 (PMC6978187; doi:10.1016/j.eclinm.2019.10.011)
Supplement: Supplementary file 1 [file mmc1.doc]

STROBE Statement—Checklist of items that should be included in reports of ***cross-sectional studies***

|  | Item No | Recommendation | Author’s response |
| --- | --- | --- | --- |
| **Title and abstract** | 1 | (*a*) Indicate the study’s design with a commonly used term in the title or the abstract | Done.  The title indicates it’s a quasi-experimental study |
|  |  | (*b*) Provide in the abstract an informative and balanced summary of what was done and what was found | Done.  Mentioned in the abstract. |
| Introduction | | |  |
| Background/rationale | 2 | Explain the scientific background and rationale for the investigation being reported | Done.  Explained in the introduction section. |
| Objectives | 3 | State specific objectives, including any prespecified hypotheses | Done.  Specified in the last paragraph of introduction section. |
| Methods | | |  |
| Study design | 4 | Present key elements of study design early in the paper | Done.  Mentioned in the section on evaluation approach. |
| Setting | 5 | Describe the setting, locations, and relevant dates, including periods of recruitment, exposure, follow-up, and data collection | Done.  Intervention and control settings have been described separately, and also presented in Table 1. The study location is also mentioned under a separate sub-heading. Details of data collection is presented in the third paragraph under evaluation approach. |
| Participants | 6 | (*a*) Give the eligibility criteria, and the sources and methods of selection of participants | Done.  Mentioned under the section on study population and evaluation approach. |
| Variables | 7 | Clearly define all outcomes, exposures, predictors, potential confounders, and effect modifiers. Give diagnostic criteria, if applicable | Done.  Mentioned in the measures section. |
| Data sources/ measurement | 8* | For each variable of interest, give sources of data and details of methods of assessment (measurement). Describe comparability of assessment methods if there is more than one group | Done.  Mentioned in the measures section. |
| Bias | 9 | Describe any efforts to address potential sources of bias | Done.  Mentioned in the third paragraph under discussion section. |
| Study size | 10 | Explain how the study size was arrived at | Done.  Mentioned in the section on study location and population. |
| Quantitative variables | 11 | Explain how quantitative variables were handled in the analyses. If applicable, describe which groupings were chosen and why | Done.  Explained under the measures section. |
| Statistical methods | 12 | (*a*) Describe all statistical methods, including those used to control for confounding | Done.  Mentioned in the section on statistical analysis. |
|  |  | (*b*) Describe any methods used to examine subgroups and interactions | No separate analysis on subgroups/ interactions was done. |
|  |  | (*c*) Explain how missing data were addressed | No missing data. |
|  |  | (*d*) If applicable, describe analytical methods taking account of sampling strategy | Done.  Mentioned in the section on statistical analysis. |
|  |  | (*e*) Describe any sensitivity analyses | No sensitivity analysis was done. |
| Results | | |  |
| Participants | 13* | (a) Report numbers of individuals at each stage of study—eg numbers potentially eligible, examined for eligibility, confirmed eligible, included in the study, completing follow-up, and analysed | Done.  Refer to Figure 2. |
|  |  | (b) Give reasons for non-participation at each stage |  |
|  |  | (c) Consider use of a flow diagram | Done. Figure 2. |
| Descriptive data | 14* | (a) Give characteristics of study participants (eg demographic, clinical, social) and information on exposures and potential confounders | Done.  Mentioned in the first paragraph under results section and in Table 2. |
|  |  | (b) Indicate number of participants with missing data for each variable of interest | No missing data. |
| Outcome data | 15* | Report numbers of outcome events or summary measures | Done.  Presented in the second paragraph of results section and in Table 3. |
| Main results | 16 | (*a*) Give unadjusted estimates and, if applicable, confounder-adjusted estimates and their precision (eg, 95% confidence interval). Make clear which confounders were adjusted for and why they were included | Done.  Presented in the second and third paragraph under results section, and in Table 3 and Table 4. |
|  |  | (*b*) Report category boundaries when continuous variables were categorized | Done. |
|  |  | (*c*) If relevant, consider translating estimates of relative risk into absolute risk for a meaningful time period | Not applicable. |
| Other analyses | 17 | Report other analyses done—eg analyses of subgroups and interactions, and sensitivity analyses | No subgroups/ interactions/ sensitivity analyses were carried out. |
| Discussion | | |  |
| Key results | 18 | Summarise key results with reference to study objectives | Done.  Summarized in the first paragraph of Discussion section. |
| Limitations | 19 | Discuss limitations of the study, taking into account sources of potential bias or imprecision. Discuss both direction and magnitude of any potential bias | Done.  Mentioned in the third paragraph under the discussion section. |
| Interpretation | 20 | Give a cautious overall interpretation of results considering objectives, limitations, multiplicity of analyses, results from similar studies, and other relevant evidence | Done.  Mentioned in the last paragraph under the discussion section. |
| Generalisability | 21 | Discuss the generalisability (external validity) of the study results | Done.  Mentioned in the last paragraph under the discussion section. |
| Other information | | |  |
| Funding | 22 | Give the source of funding and the role of the funders for the present study and, if applicable, for the original study on which the present article is based | Done.  The funding source is mentioned under acknowledgement. Role of two authors, who were from funders and provided technical insights in the sampling design, is mentioned under the heading ‘role of funding source’. |

*Give information separately for exposed and unexposed groups.

**Note:** An Explanation and Elaboration article discusses each checklist item and gives methodological background and published examples of transparent reporting. The STROBE checklist is best used in conjunction with this article (freely available on the Web sites of PLoS Medicine at http://www.plosmedicine.org/, Annals of Internal Medicine at http://www.annals.org/, and Epidemiology at http://www.epidem.com/). Information on the STROBE Initiative is available at www.strobe-statement.org.
